# Unwell in the system: Ethical blind spots and structural injustices in modern psychiatry—Toward a humanistic renewal

**DOI:** 10.1371/journal.pmen.0000415

**Published:** 2025-08-29

**Authors:** Mohit Kumar, Sanjay Kumar, Khusboo, Vinit Kumar Singh, Amit Kumar Soni, Rounak Chatterjee

**Affiliations:** 1 Department of Psychiatry, All India Institute of Medical Sciences, Bhopal, India; 2 Department of Clinical Psychology, Institute of Mental Health Amritsar, Amritsar, Punjab, India; 3 Department of Psychiatry, Chirayu Medical College & Hospital, Bhopal, India; 4 Department of Administration, All India Institute of Medical Sciences, Bhopal, India; 5 Department of Psychology, Government MLB Girls PG College, Kila Bhawan, Devi Ahilya University, Indore, India; PLOS: Public Library of Science, UNITED KINGDOM OF GREAT BRITAIN AND NORTHERN IRELAND

Psychiatry, as both a medical discipline and sociocultural institution, stands at a complex intersection of science, society, and suffering. Despite its mission to relieve mental distress, psychiatry has often relied on frameworks that marginalize patients’ voices and oversimplify the root causes of suffering [1]. The biomedical model, while scientifically robust, tends to neglect trauma, poverty, racism, and other structural determinants of health. As psychiatry aligns itself more closely with neuroscience and pharmacology, it risks distancing itself from the ethical imperatives of patient-centered, holistic care.

## Diagnostic overshadowing and epistemic injustice

The dominance of categorical diagnoses in systems like the DSM and ICD enables important standardization, facilitates research, and provides clinicians with a common language. However, this utility often comes at the cost of individual nuance [2]. Diagnostic overshadowing, where clinicians overly rely on a diagnosis and overlook a patient’s lived experience, can perpetuate epistemic injustice. This form of injustice, as defined by philosopher Miranda Fricker, occurs when individuals are discredited as knowers of their own experiences [3].

For marginalized populations, whose expressions of distress often diverge from normative expectations, psychiatric categorization can become a silencing force rather than a healing tool. While diagnoses can be useful for triaging care or coordinating services, they must be used with humility and flexibility. Clinicians should remain open to patients’ narratives that may challenge or expand upon diagnostic frameworks.

## Involuntary treatment and coercion

Coercive practices such as involuntary hospitalization and forced medication remain ethically fraught, though in certain circumstances such as when patients pose an imminent risk to themselves or others, they may be necessary to prevent harm. Emergency interventions can save lives and stabilize severe psychiatric episodes, particularly when individuals lack insight or are in acute crisis [4].

Nonetheless, these measures challenge the foundational principle of autonomy and can have lasting negative effects on patient trust and engagement with mental health services. Moreover, studies reveal disparities in how coercion is applied, with racial and socioeconomic biases influencing decisions a clear violation of justice and equity in care [5]. Ethical psychiatry must prioritize trauma-and culture-informed, consent-centered alternatives wherever possible, such as supported decision-making and advance directives [6].

## Neglect of social determinants and structural violence

Perhaps the most glaring ethical omission is psychiatry’s failure to address social determinants of mental health. Factors such as housing insecurity, systemic racism, community violence, and unemployment are well-established contributors to psychological distress, yet they are rarely addressed in psychiatric settings [7]. The concept of structural competence teaching clinicians to recognize and respond to upstream social causes offers a path forward [8]. Programs in global health, social psychiatry, and trauma-informed care demonstrate that integrating social context can significantly improve outcomes.

## Internal stigma and professional implications

Paradoxically, while psychiatry champions the destigmatization of mental illness in patients, it often stigmatizes mental health issues among its own professionals. Many psychiatrists face intrusive licensing questions about their mental health history, which can deter them from seeking help [9,10]. Despite calls by the American Medical Association and the Federation of State Medical Boards to reform such practices [11], inconsistencies persist across jurisdictions. This internal stigma undermines both clinician well-being and patient safety and sends a dangerous message: that mental illness is incompatible with professional competence [12].

## Pharmaceutical influence and commercialization

The relationship between psychiatry and the pharmaceutical industry is complex and often problematic. Many drug trials are funded by pharmaceutical companies, raising concerns about selective reporting, inflated efficacy claims, and underreporting of adverse effects [13]. This commercialization of care has led to the medicalization of ordinary human emotions, while overshadowing non-pharmacological interventions like psychotherapy, peer support, and community-based care. Ethical psychiatry must reclaim its commitment to balanced, evidence-informed treatment options.

## Toward ethical renewal: Rehumanizing psychiatry

To correct these blind spots, psychiatry must re-center itself on ethical and humanistic values. This involves embracing narrative medicine, which values patient stories; adopting trauma-informed care, which recognizes the impact of past trauma on present behavior; and implementing culturally sensitive practices that respect diverse worldviews. Globally, models like the Open Dialogue approach in Finland which emphasizes immediate and inclusive therapeutic conversations involving patients, their families, and clinicians from the onset of a crisis, promoting collaborative decision-making and mutual respect. Similarly, community mental health networks in India and Latin America embody culturally rooted and socially integrated models of care, focusing on community participation, holistic interventions, and addressing socio-environmental determinants of mental health. [14]. Moreover, actively involving service users in policy formulation, research, and clinical decision-making helps ensure psychiatric practices remain grounded in real-world, lived experiences, enhancing their relevance, fairness, and effectiveness [15].

## Future directions

To bridge the ethical blind spots identified in this article, several key areas warrant further development and empirical attention:

1. **Integrating Structural Competence into Training**

Psychiatry training programs should formally incorporate structural competency, equipping clinicians to identify and act upon the social and economic determinants of health. Metzl and Hansen advocate for this shift to empower clinicians in addressing upstream factors contributing to mental distress [9].

2. **Expanding Community-Based Care**

Future models must prioritize decentralization and community ownership of mental health services. Evidence from Brazil’s *Centros de Atenção Psicossocial* (CAPS) and India’s *District Mental Health Programme* (DMHP) suggests that such community-based models can reduce stigma and enhance accessibility [8].

3. **Ethical Review of Coercion Practices**

Research should continue to explore alternatives to involuntary treatment, such as supported decision-making, advance directives, and crisis respite centers. Implementation studies could evaluate how trauma-informed and consent-centered approaches impact outcomes in acute psychiatric care [6].

4. **Promoting Lived Experience Leadership**

Involving service users not just as patients but as co-researchers, peer mentors, and policy advocates is essential. Participatory approaches can reduce epistemic injustice and ensure systems reflect the real-world needs of users [13].

5. **Global South-Centered Frameworks**

Psychiatry must resist one-size-fits-all biomedical models and elevate healing practices rooted in local cultural, spiritual, and community traditions. The Movement for Global Mental Health calls for pluralistic and rights-based approaches, especially in low- and middle-income contexts [14].

## Conclusion

Psychiatry stands at a pivotal crossroads. While it has achieved notable scientific milestones in neurobiology and pharmacology, its ethical and humanistic foundations have not evolved at the same pace. Addressing persistent blind spots such as diagnostic reductionism, coercive practices, commercial bias, neglect of social determinants, and internal stigma is not merely a matter of clinical refinement but of moral urgency.

A psychiatry that pathologizes distress without acknowledging context, that prioritizes control over consent, or that silences its own practitioners when they suffer, risks becoming part of the problem rather than the solution. We must therefore move beyond symptom containment toward systems that restore dignity, trust, and equity.

This ethical renewal demands more than individual reflection it requires institutional transformation. We call upon psychiatric associations, training institutions, healthcare systems, and policymakers to embed structural competence, trauma-informed care, and lived experience leadership into the very architecture of mental health practice. Only by re-centering ethics, empathy, and equity can psychiatry become not just more effective, but more humane and just for all it seeks to serve.
